# Thermal Remodeling of Human HDL Particles Reveals Diverse Subspecies

**DOI:** 10.1021/jasms.4c00228

**Published:** 2024-07-25

**Authors:** Corinne A. Lutomski, Tarick J. El-Baba, David E. Clemmer, Martin F. Jarrold

**Affiliations:** † Department of Chemistry, Indiana University, 800 E. Kirkwood Ave., Bloomington, Indiana 47405, United States

## Abstract

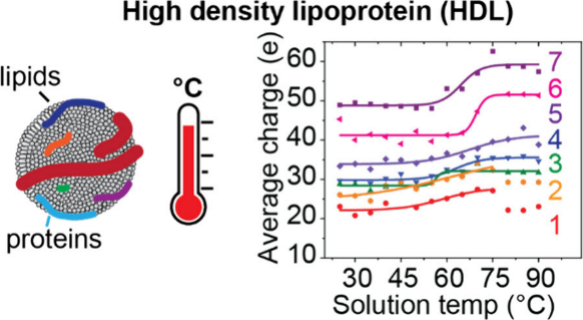

High-density lipoproteins (HDL) are micelle-like particles consisting of a core of triglycerides and cholesteryl esters surrounded by a shell of phospholipid, cholesterol, and apolipoproteins. HDL is considered “good” cholesterol, and its concentration in plasma is used clinically in assessing cardiovascular health. However, these particles vary in structure, composition, and therefore function, and thus can be resolved into subpopulations, some of which have specific cardioprotective properties. Mass measurements of HDL by charge detection mass spectrometry (CD-MS) previously revealed seven distinct subpopulations which could be delineated by mass and charge [Lutomski, C. A. et al. Anal. Chem. 2018]. Here, we investigate the thermal stabilities of these subpopulations; upon heating, the particles within each subpopulation undergo structural rearrangements with distinct transition temperatures. In addition, we find evidence for many new families of structures within each subpopulation; at least 15 subspecies of HDL are resolved. These subspecies vary in size, charge, and thermal stability. While this suggests that these new subspecies have unique molecular compositions, we cannot rule out the possibility that we have found evidence for new structural forms within the known subpopulations. The ability to resolve new subspecies of HDL particles may be important in understanding and delineating the role of unique particles in cardiovascular health and disease.

## Introduction

High-density lipoprotein (HDL) is a heterogeneous mixture of particles found in circulation that contains proteins, lipids, and other small molecules.^1^ HDL levels are directly related to cardiovascular health. HDL is constricted to a lipid micellar structure by a protein belt consisting of structural protein Apolipoprotein A1 (ApoA1), which wraps around the particle’s surface, while larger HDL particles contain varying numbers of both ApoA1 and apoliporotein A2 (ApoA2). Different ratios of lipids and proteins will produce particles with different physiochemical properties (e.g., diameter, density) which results in HDL with an array of possible subpopulations. However, only five to ten subpopulations can typically be distinguished based on physical properties alone; ultracentrifugation results in the separation of large and light particles from small, dense HDL, while further separation of individual subpopulations relies on additional analytical intervention through the use of density gradient ultracentrifugation.^2^ Alternatively, non-denaturing polyacrylamide gel electrophoresis has observed five distinct subpopulations.^3^ In addition, it is well-established that these subpopulations contain vast proteomic diversity; over 95 proteins are known to associate with HDL particles, resulting in diverse subspecies that exist within a single subpopulation.^4^ Affinity purification of HDL subspecies from plasma using antibodies against 16 of such proteins revealed that the proteomes of the subspecies differed significantly from total HDL and provided evidence for distinct functional groupings of particles involved in lipid metabolism, hemostasis, or anti-inflammatory processes.^5^ Thus, the ability to interrogate the molecular nature and physical properties of the diverse number of subspecies that exist within the heterogeneous ensemble of HDL is fundamentally important to understanding human health.^6−10^

Because individuals with increased levels have a lower risk of developing cardiovascular disease (CVD), HDL is often described as “good” cholesterol.^11^ While assessing CVD risk is valuable, therapies based on increasing HDL in at-risk individuals have mixed outcomes.^12−16^ It is believed that a better understanding of how variations in particle size, density, composition, stability, and structure will impact the understanding of how HDL influences cardiovascular health and improve CVD treatment strategies, which requires new analytical strategies which can delineate such differences in a straightforward manner.^17−26^

Previously, we investigated the mass and electrospray charging properties of HDL using charge detection mass spectrometry (CD-MS). Unlike other MS methods that determine only mass-to-charge (*m*/*z*) ratios, CD-MS simultaneously measures *m*/*z* and *z*. This is especially important when analyzing large, heterogeneous particles because the combination of measurements for each particle eliminates ambiguities when determining the intact mass. Unless one already has prior knowledge of one of the variables (*m* or *z*) neither can be inferred from the MS measurements of *m*/*z* alone.^27−33^ We found evidence for at least seven distributions of HDL particles that were distinct in their mass. These species also differed in electrospray charging properties, as larger particles retain more charge than smaller particles during the final stages of droplet desolvation during transit into the mass spectrometer. It is intriguing that enumeration of seven subpopulations by CD-MS, based solely on mass and, indirectly, the particle diameter, is similar to the five to ten subpopulations measured by other analytical strategies.^24,34^ It is not unreasonable that the abundances of these subpopulations measured by CD-MS could provide a rapid readout of cardiovascular heath, similar to preclinical and approved diagnostic tests. However, it is still not clear if a more diverse array of subpopulations with different protein–lipid ratios and proteomes are present within each mass distribution.

One avenue for understanding subpopulations involves monitoring differences in their thermal stabilities. It has become increasingly common to couple variable-temperature ESI (vT-ESI) to MS to evaluate melting temperatures (*T*_m_s) of protein folds,^35,36,42,43^ protein assemblies,^37,44^ and protein–ligand complexes.^38,39^ In vT-ESI-MS, thermal stabilities are determined by measuring shifts in ESI charge state distributions or ion mobility collision cross sections, which report on equilibrium populations of folded/unfolded, assembled/disassembled, and ligand-bound/apo states, respectively. Comparatively, HDL is an incredibly complex particle. However, MS has matured to a state in which biophysical measurements can be carried out directly from heterogeneous mixtures of proteins and small molecules,^40^ cell lysates,^41^ and even native membranes.^42,43^ Therefore, we hypothesized that vT-ESI and CD-MS could be used to detect differences in temperature-dependent charging properties within the HDL subpopulations, thereby providing an avenue to identify new features based on their intrinsic thermal stabilities/ *T*_m_s. In exploring this hypothesis, we found evidence for at least 15 subspecies – HDL particles with distinct thermal properties that share similar overall masses to the individual subpopulations.

## Results and Discussion

**Figure 1:**
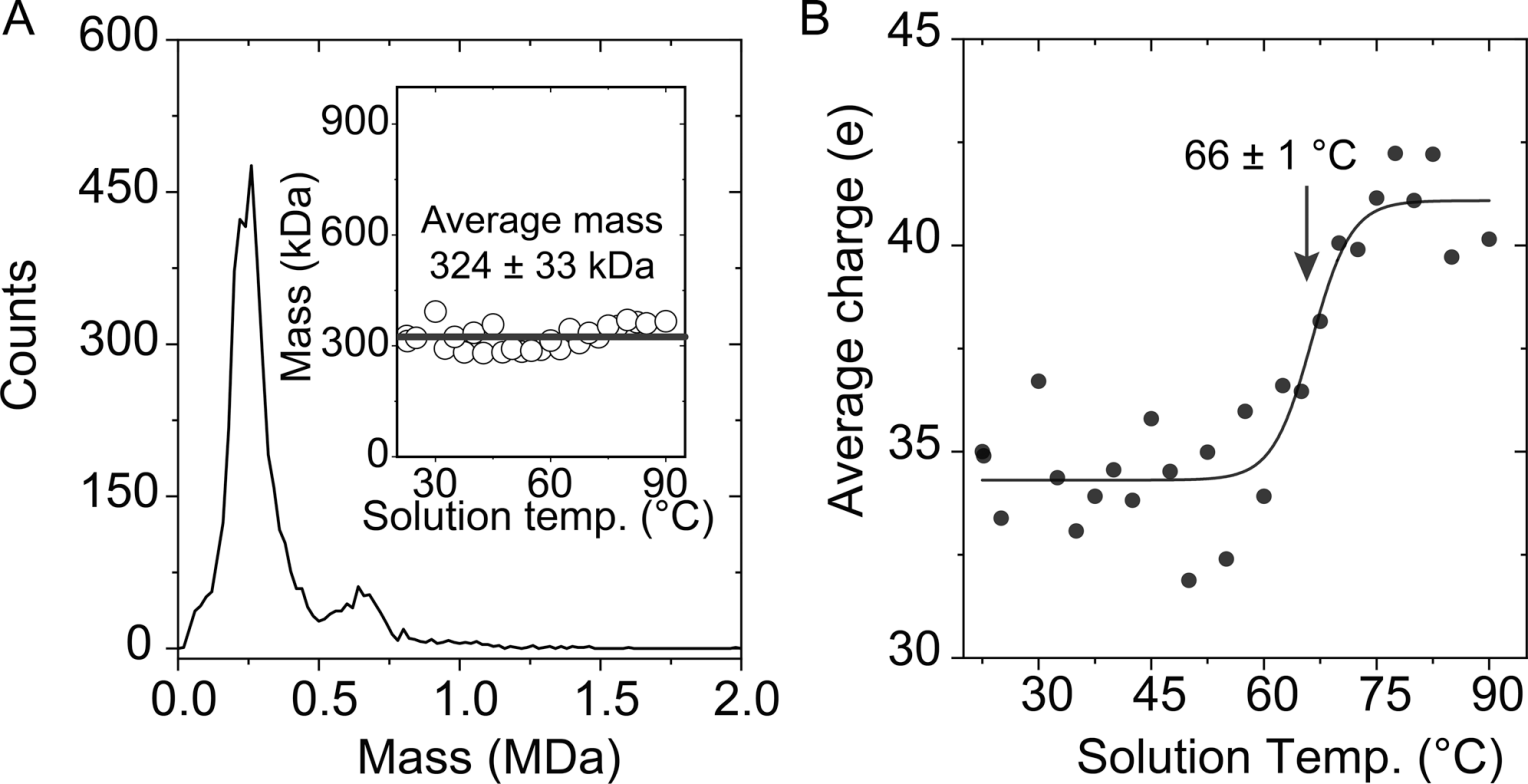
Ensemble measurement of HDL by CD-MS. (A) shows a CD-MS mass spectrum of purified HDL from human plasma (∼3 μM solution in 10 mM NH_4_OAc, pH = 7.7). The spectrum was generated using 50 kDa bins. Open symbols show the average mass of HDL at each solution temperature and the dashed gray line at 324 kDa represents the average mass of HDL across all temperatures. (B) shows the average charge state as a function of solution temperature. HDL transitions from low to high charge at a midpoint temperature (*T*_m_) of 66 ± 1 °C.

We generated a CD-MS mass spectrum of HDL, electrosprayed from a pH 7.7 solution maintained at 25 °C (Figure 1). While small populations of ions extended to ∼1 MDa, the spectrum was dominated by a broad feature at ∼243 kDa, and a smaller shoulder, centered at ∼650 kDa. Data recorded for solutions at elevated temperatures led to mass spectra that were very similar to the data shown in Figure 1, where the average mass remained constant at ∼324 ± 33 kDa within measurement uncertainty, over the entire 22 to 90 °C temperature range (Figure 1**A inset**). These two observations (i. e., that the mass spectrum at each temperature was dominated by a peak at ∼243 kDa; and, that the average mass for all particles did not change with temperature) indicated that in this solution, HDL did not dissociate into smaller particles, or coalesce to form larger species over the tested temperature range. Despite no changes in overall mass, the average charge for all ions underwent a drastic change across solution temperatures from 22 to 90 °C. Below ∼55 °C the charge was relatively constant, at *z* ∼ 34 *e*, while at temperatures above ∼55 °C the average charge increased abruptly to a value of *z* ∼ 42 *e* where it then remained. The sigmoidal shape for the average charge with respect to temperature reveals a transition midpoint, *T*_m_, (viz. melting temperature when analytes are undergoing a phase transition) is 66 ± 1 °C, in agreement with *T*_m_ = 65 °C for HDL solutions (pH 7.7) determined from circular dichroism measurements.^44^ The circular dichroism measurements showed a second transition at ∼89 °C which has also been observed in electron microscopy studies, and is assigned to high-temperature fusion of HDL particles.^20^ Above ∼90 °C, the solution became turbid and we could not maintain a stable ESI signal. We attributed this effect to coalescence of particles which resulted in insoluble aggregates in what appears to be an irreversible process (Figure S1).

**Figure 2:**
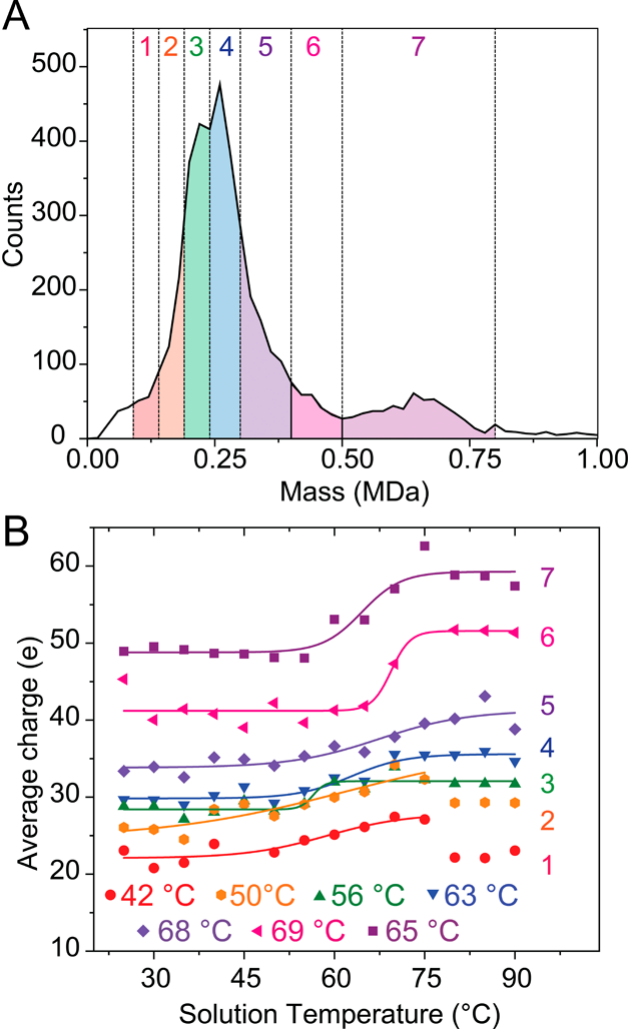
Resolution of HDL into seven subpopulations. The mass spectrum in (A) shows the mass boundaries for each subpopulation (represented by colored peaks) relative to the total HDL mixture (black trace). Ions for these subpopulations are colored in red, orange, green, blue, violet, pink, and purple and correspond to subpopulations one through seven, respectively. (B) Average charge state as a function of solution temperature for all seven subpopulations. Legend shows midpoint transition temperatures for each subpopulation.

An advantage of CD-MS is that there are no ambiguities due to overlapping charge states between subpopulations. In CD-MS, both *m*/*z* and z are measured, and therefore HDL could be grouped into subpopulations based on their intact mass and which were defined previously.^45^ By using well-defined mass ranges which do not overlap (Figure 2A), the ions comprising each subpopulation are therefore distinct, which allowed us to track structural changes reflected in the charge state, even when the charge states are similar across different subpopulations. The sigmoidal curves representing thermal transitions have midpoints which report on unique *T*_m_ values. When heated, the average charge of each subpopulation remains relatively constant and then abruptly increases. For example, the largest, most massive species (subpopulation 7 centered at *m* ∼ 618 kDa) increases in charge from *z* ∼ 48 to ∼58 *e* with a *T*_m_ = 65 ± 2 °C. Subpopulation 6 (the second highest mass subpopulation, centered at ∼440 kDa) displays similar behavior, increasing in charge from *z* ∼ 41 to *z* ∼ 51 *e* with a midpoint of *T*_m_ = 69 ± 1 °C. Overall, the midpoint temperatures of subpopulations varied substantially (from *T*_m_ ∼ 42 to 69 °C), indicating that the stabilizing components (e.g., protein compositions) differ.

**Figure 3:**
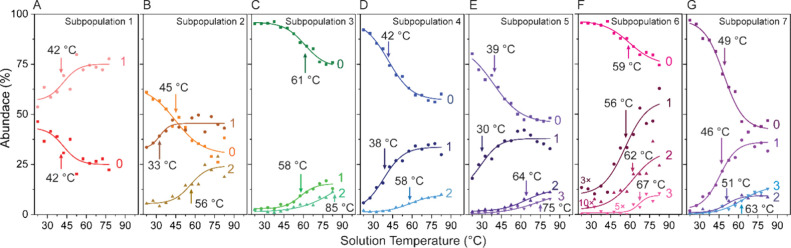
Emergence of high charge state products for subpopulations 1 through 7 (A through G) at elevated solution temperatures. For simplicity, we label the product ions with the average charge following the grouping of charge windows. Legend depicts transition temperatures for each subspecies.

In previous experiments studying the unfolding of proteins, it was possible to simultaneously follow the loss of precursors, the formation of intermediates, and finally the presentation of thermal products with increases in temperature.^46,47^ We reasoned that the subpopulations measured here may present with similar “intermediate” subspecies, however such intermediates may instead reveal differences in protein and lipid composition which give rise to unique thermal transitions. Across all seven subpopulations, the average charge only slightly increases, Δ*z̅* ∼ 10 e and a ∼ 5e increase overall. It is important to consider that the major protein component of HDL, ApoAI, wraps around the lipid monolayer that is comprised of positively and negatively charged phospholipids. In dilute solution, it is reasonable to expect that the unfolding of ApoAI would lead to behavior we observed previously, as in, the exposure of ionizable residues upon heating should lead to large increases in charge. Interestingly, we do not observe such behavior here, presumably because the surface lipids in contact with ApoAI and the aqueous solution are good proton scavengers, so increases in charge due to ApoAI/HDL remodeling are expected to be subtle. It may also be that structural changes are subtle and do not materialize as large shift in charge state.^48^ As each subpopulation is comprised of a heterogeneous mixture of particles with different protein and lipid composition, it becomes challenging to track the transition into distinct product states using discrete charge states. Instead, we explored the hypothesis that subpopulations may transition into different product states which are reflected by broad changes in *m*/*z*. As CD-MS measures individual ions, we were able to count individual particles that emerge within a range of *m*/*z* for a given subpopulation at each temperature sampled. Similar to our previous approaches, we grouped product species that showed identical thermal transition profiles and similar formation *T*_m_s.^42^ After manually iterating over multiple window sizes (Figures S2–8), the resulting plots of the normalized abundance of the precursors (0) and their respective products (1, 2 or 3) as a function of temperature is shown in Figure 3. Subpopulation 1, which is expected to have the least diversity in its proteome, exhibits two-state behavior where only a single product emerges. For Subpopulations 2–7, multiple products can be observed (Figure 3**B-G**). Notably, for these subpopulations, a low temperature product (1) and at least one high temperature product appear, giving further credence to the hypothesis that the stabilizing protein components differ. For subpopulations of increasing mass (e.g., subpopulations 5 through 7), and therefore compositional heterogeneity, we find evidence for up to three distinct subspecies (Figure 3**E-G**). Interestingly, we find little correlation between mass and stability; the precursor subspecies of subpopulation 3 appears to be the most stable with a *T*_m_ of 61 °C, and remains >75% abundant across the entire temperature range. Meanwhile, many subspecies within a larger subpopulation, such as in subpopulation 5, appear over a range of temperatures, and the precursor population is reduced to <50% of the total signal. This behavior suggests some of the subspecies may emerge from a series of sequential structural transitions in one related subspecies of HDL.

When combined, these ideas provide evidence that the new high temperature features arise when distinct subspecies that are present, but unresolved and therefore hidden at low temperatures, undergo unique thermal transitions that enable them to be resolved. In total, the signals for the seven subpopulations evolve into 15 subspecies.

As HDL subpopulations are extremely heterogeneous with vastly diverse proteomes and lipidomes,^49^ we propose that the elevated temperatures facilitate ample opportunity for structural changes due to alteration in the protein conformation as well as restructuring of the lipid phases,^50^ however, both are currently hidden to the current approach.

## Conclusions

We explored the extent that HDL subpopulations could be resolved by coupling vT-ESI with CD-MS. Based on unique thermal transitions and mass, we found evidence for at least 15 subspecies present within the seven previously defined HDL subpopulations. In the context of emerging studies using immunoprecipitation-MS to resolve subspecies based on proteomic content, this is a conservative estimate of the true number of HDL subspecies that exist within human HDL.^1−3^ The thermal transitions for similarly sized HDL assemblies that are described below reveal that subspecies differ in charge–but, not mass. This leads us to propose that these new subfamilies differ either by the nature of the proteins on the surface of the particles, or are associated with the unique structural forms that are favored at elevated temperatures. The ability to characterize and study not only subpopulations, but subspecies within those subpopulations in HDL with mass-spectrometric precision will complement biochemical analyses aimed at understanding the roles of different HDL species in cardiovascular health.

We cannot resist suggesting possible origins of the thermal transition behavior we observe for different subpopulations and their corresponding subspecies. The outer phospholipids of HDL are stabilized by an apolipoprotein armature that wraps around the particle. The stoichiometry and composition of this framework is highly variable and more than 95 different HDL proteins have been identified.^51,52^ The smallest HDL subpopulation (1) has the lowest transition temperature. Smaller particles are expected to have fewer apolipoproteins and thus should be less stable. This idea is consistent with the relatively low value of *T*_m_ = 42 °C. The larger-sized subpopulations 2 to 6 are more stable with *T*_m_ > 50 °C. Presumably this is because larger particles incorporate additional proteins into the stabilizing scaffold. An exception is the most massive, ∼ 618 kDa subpopulation (7), where *T*_m_ = 65 °C is significantly lower than *T*_m_ = 68 and 69 °C for subpopulations 5 and 6. This result suggests that the composition of subpopulation 7 differs from the other subpopulations. It is well-known that large HDL incorporates apolipoprotein E resulting in an enhanced capacity to carry cholesterol, creating large, lipid-rich particles.^53−55^ Akin to calorimetric measurements of LDL, where the melting temperature correlated inversely with the cholesterol and triglyceride lipid ratios,^56^ we propose that the lower *T*_m_ of subpopulation 7 is consistent with a significant difference in apolipoprotein composition and likely contains more lipid by mass. Finally, one of the subspecies in subpopulation 5 exhibits unique thermal behavior where the charge increases but then decreases again beyond a temperature threshold around 60 °C (Figure 3E). There are two likely explanations: (i) aggregation or dissociation of highly charged particles which then causes a drop-out of signal and thus decreases the overall charge of the ions remaining in that subspecies, (ii) or the subspecies undergoes a drastic rearrangement and overall collapse in the structure, where the smaller and more compact particle size manifests as a lower overall charge. In the absence of ion mobility, we can only speculate that there may be conformational changes to the proteins on the surface of HDL, similar to previous observations.^57^ Aggregation can be ruled out due to the absence of high molecular weight (>1 MDa) ions at these temperatures.

Finally, within a subpopulation, resolved subspecies exist over a common range of masses; however, they have unique appearance temperatures and differ in *m*/*z*. Decreases in *m*/*z*, or increases in charge between adjacent subspecies, suggest that an additional protein unit (that is partially exposed at the subspecies’ surface) is accessible. This would be consistent with a structural transition where upon heating a single subpopulation undergoes multiple structural transitions with increasing temperature. Each transition may expose regions of the protein framework to the surface, resulting in differences in mass-to-charge ratio. Alternatively, a single subpopulation might be comprised of subspecies having different compositions–but the same mass. In this case the more highly charged subspecies may have additional proteins incorporated into the supporting armature. This not only accounts for the apparent variations in *m*/*z* of each subspecies, but also the observation of a systematic increase in the temperature required to resolve each subspecies, which is consistent with increased stability.

## Experimental Section

A complete description of the experimental details is provided in the Supporting Information. A complete description of the CDMS instrument and vT-ESI apparatus used here are provided elsewhere.^29,42,43^ HDL was purchased from Academy Bio-Medical Company (Houston, TX), and buffer exchanged into 10 mM ammonium acetate prior to analysis. In low ionic strength buffers from pH 5.7–7.7, HDL are highly thermostable, and increases in salt concentrations up to 300 mM have been shown to shift calorimetric transitions to lower temperatures by as much as 14 °C.^44^ Furthermore, increased ionic strength (150 mM NaCl) has been shown to accelerate thermal-induced particle aggregation of low-density lipoproteins.^58^ Because these experiments required a stable electrospray for prolonged periods over a wide range of temperatures, and the extraction of useful information (e.g., transition temperature) is sensitive to protein aggregation, we opted to use low ionic strength solution conditions.10–30 μL of sample was loaded into borosilicate capillaries prepared in house, which were positioned 1–3 mm from the orifice of the CDMS instrument. An electrospray was generated by applying a ∼ 1 kV potential to a platinum wire inserted into the capillary and immersed in the analyte solution. Data were processed offline using OriginPro.

## Supporting Information Available

Experimental details, temperature-dependent CD-MS spectra for each subpopulation, reversibility of thermal transitions, elimination of the possibility of particle aggregation/fusion (PDF)
