# Supplementary material for: Thermal Remodeling of Human HDL Particles Reveals Diverse Subspecies
Source: J Am Soc Mass Spectrom. 2024 Jul 25;35(8):2002–7. doi: 10.1021/jasms.4c00228 (PMC11311237; doi:10.1021/jasms.4c00228)
Supplement: Supplementary file 1 — js4c00228_si_001.pdf [file js4c00228_si_001.pdf]

Supporting Information for:

Thermal Remodeling of Human HDL Particles Reveals Diverse Subspecies

Corinne A. Lutomski,[1,2,*] Tarick J. El-Baba,[1,2,*] David E. Clemmer,[1,*] and Martin F. Jarrold[1,*]

1. Department of Chemistry, Indiana University, 800 E. Kirkwood Ave., Bloomington IN 47405, USA
2. Present Address: Kavli Institute for Nanoscience Discovery and Department of Chemistry, Dorothy Crowfoot Hodgkin Building, University of Oxford, OX1 3QU, Oxford, UK

Correspondence to: corinne.lutomski@chem.ox.ac.uk, tarick.el-baba@chem.ox.ac.uk,

mfj@indiana.edu, clemmer@indiana.edu

Experimental

Sample Preparation

Purified high-density lipoprotein (HDL) from human plasma purchased from Academy Bio-Medical Company (Houston, TX). HDL was buffer exchanged into 10 mM ammonium acetate (pH 7.7) via micro Bio-Spin columns (Bio-Rad Laboratories, Hercules, CA). In low ionic strength buffers from pH 5.7-7.7, HDL are highly thermostable, and increases in salt concentrations up to 300 mM have been shown to shift calorimetric transitions to lower temperatures by as much as 14 °C.[1] Furthermore, increased ionic strength (150 mM NaCl) has been shown to accelerate thermal-induced particle aggregation of low-density lipoproteins.[2] Because these experiments required a stable electrospray for prolonged periods over a wide range of temperatures, and the extraction of useful information (e.g. transition temperature) is sensitive to protein aggregation, we opted to use low ionic strength solution conditions.

Variable Temperature (vT) nano-Electrospray Ionization (nESI)

The HDL solution was loaded into borosilicate glass electrospray capillaries (1.5 mm OD x 0.78 mm ID, filamented) pulled to ~1 µm inner diameter using a Sutter Instruments P97 Flaming/Brown micropipette puller. The capillary was placed into a home-built variable temperature nESI source described elsewhere.[3] The source design and circuit were based off previous designs.[4,5,6]

Charge Detection Mass Spectrometry

Briefly, ions were generated by nano-electrospray ionization (~1 kV) and transferred into the CD-MS instrument through a heated capillary and focused through an ion funnel. Ions are then thermalized in an RF hexapole where a DC offset on the hexapole sets the nominal ion energy at 100 eV/charge. The ions then traverse an RF-only quadrupole, and focused by an asymmetric einzel lens before entering a dual-hemispherical energy analyzer. Energy selected ions are then focused into an electrostatic ion trap that contains the charge detection cylinder. To initiate a trapping event, a potential is placed on the rear end cap so that ions are reflected through the trap. After a short delay, a potential is placed on the front end cap to close the trap, causing the ions to oscillate through the charge detection cylinder. A trapping period of 100 ms was employed, after which the trap was opened (both end caps set to ground) and the trapping cycle repeated. The ion oscillating through the detection cylinder induces a periodic signal, which is amplified, digitized, and then analyzed using fast Fourier transforms. The m/z is derived from the fundamental frequency and the charge is derived from the magnitude of the fundamental and first harmonic.

Data Processing and Visualization

Mass distributions are generated by multiplying the m/z and charge measured for each ion and binning the resulting masses. The thermal behavior of individual subpopulations is tracked by taking the average charge of all ions within a defined mass window.

The data was analyzed and visualized using OriginPro 2016 (OriginLab Corporation, Northampton, MA). Sigmoidal fits to the change in charge were generated using the non-linear curve fitting tool using a user-defined equation:

$$y(T) = y_{max} + \frac{y_{min} - y_{max}}{1 + exp\frac{(T - T_m)}{dT}} \quad \textbf{(1)}$$

where y_{max} and y_{min} are the maximum and minimum y-values. T_m is the midpoint of the transition temperature of the subpopulation which can be visualized as the inflection point in the sigmoidal fitting curve.

Supporting Figures

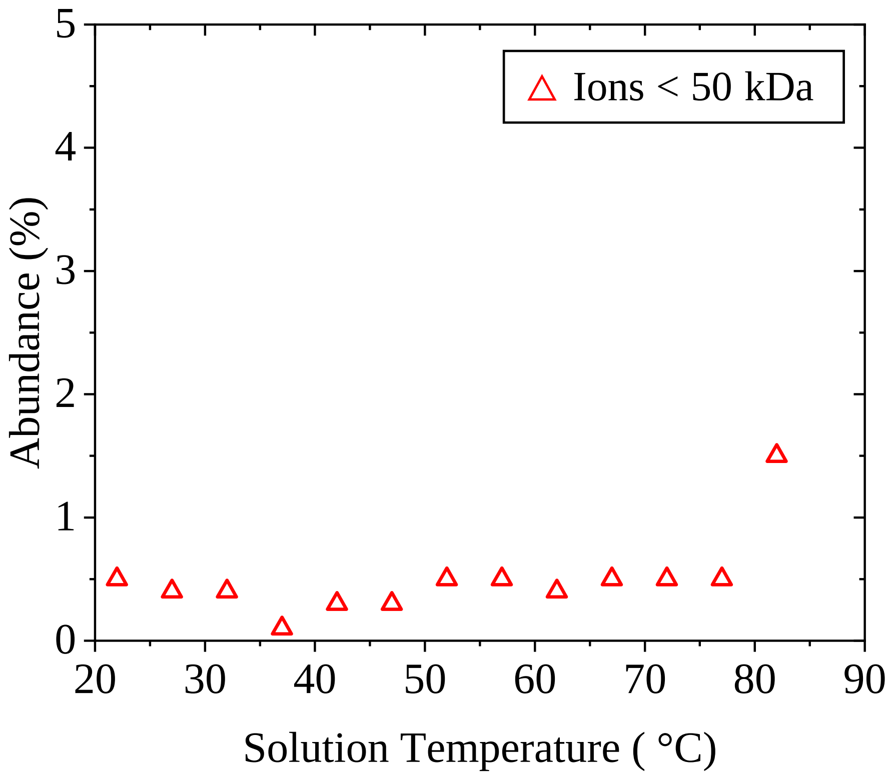

Figure S1. Plot showing the percent abundance of all ions with masses below 50 kDa with respect to solution temperature. The mass range of 0 – 50 kDa encompasses all free apolipoproteins; apo A-I and apo A-II have molecular weights of 28 and 17 kDa, respectively. The number of ions <50 kDa in mass is approximately 0.5% at all solution temperatures below 80 °C. Above 80 °C, the abundance of free apolipoprotein increases to 2%, indicating that no appreciable quantity of apolipoprotein dissociation or particle rupture occurs. Above ~90 °C, the solution became turbid and we could not maintain a stable ESI signal.

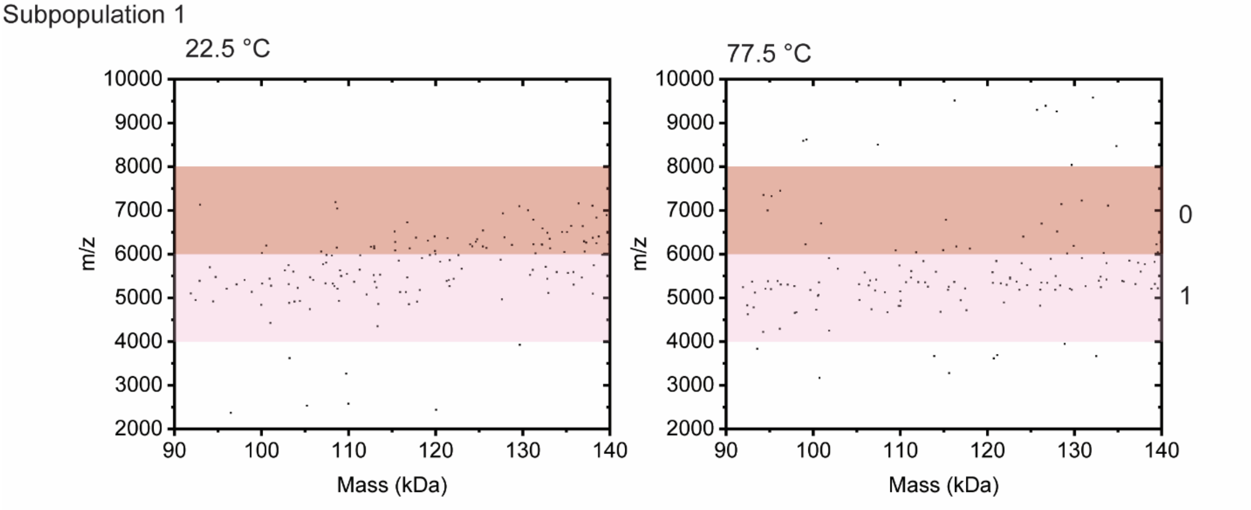

Figure S2. Scatter plot showing the mass-to-charge ratio (m/z) versus mass for every ion within subpopulation 1 at representative temperatures of (A) 22.5 °C and (B) 77.5 °C. Each point represents a single ion. Colored boxes represent m/z ranges for precursor (0, m/z 6000-8000) and product (1, m/z 4000-6000).

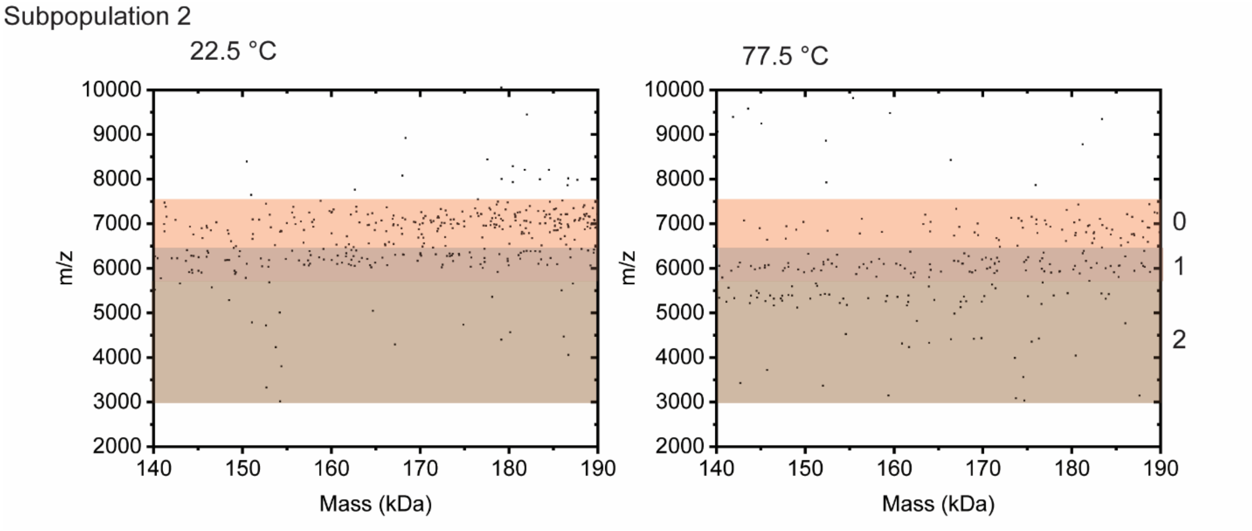

Figure S3. Scatter plot showing the mass-to-charge ratio (m/z) versus mass for every ion within subpopulation 2 at representative temperatures of (A) 22.5 °C and (B) 77.5 °C. Each point represents a single ion. Colored boxes represent m/z ranges for precursor (0, m/z 6500-7500), product 1 (m/z 5700-6500), and product 2 (m/z 3000-5700).

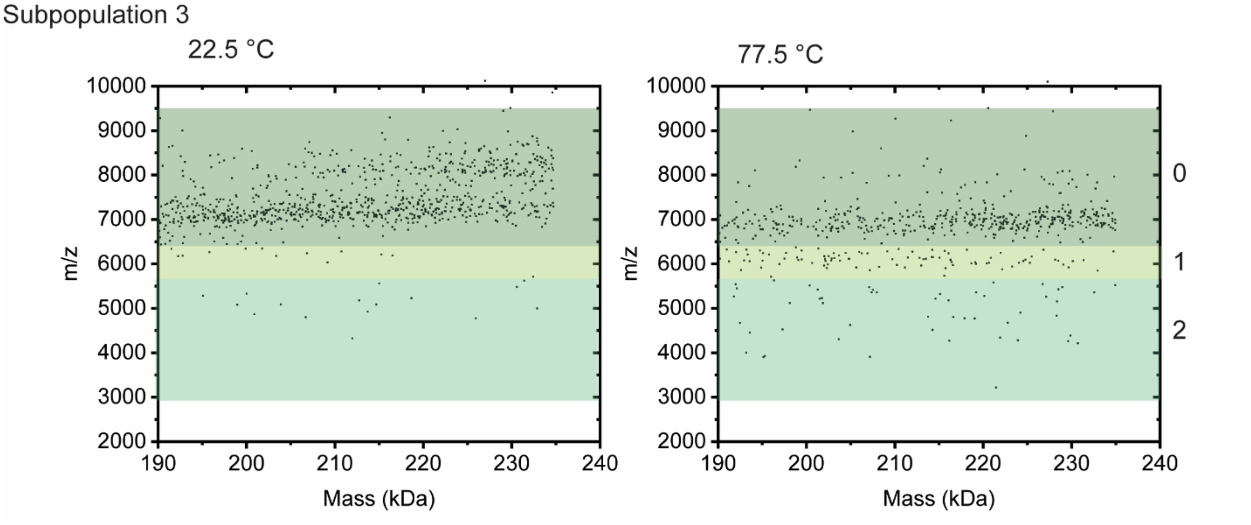

Figure S4. Scatter plot showing the mass-to-charge ratio (m/z) versus mass for every ion within subpopulation 3 at representative temperatures of (A) 22.5 °C and (B) 77.5 °C. Each point represents a single ion. Colored boxes represent m/z ranges for precursor (0, m/z 6500-9500), product 1 (m/z 5500-6500), and product 2 (m/z 3000-5500).

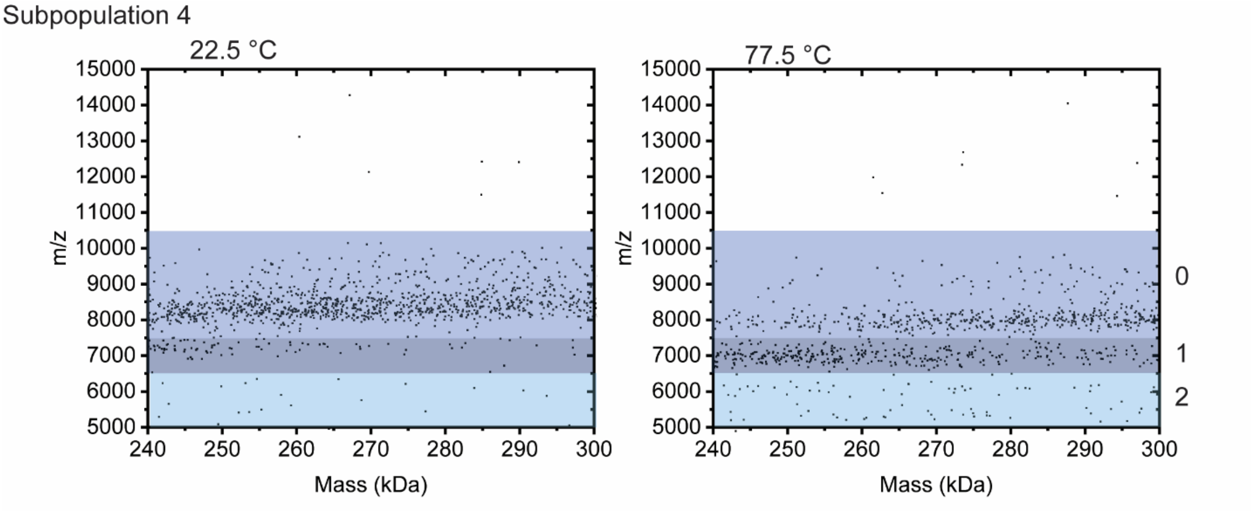

Figure S5. Scatter plot showing the mass-to-charge ratio (m/z) versus mass for every ion within subpopulation 4 at representative temperatures of (A) 22.5 °C and (B) 77.5 °C. Each point represents a single ion. Colored boxes represent m/z ranges for precursor (0, m/z 7500-10500), product 1 (m/z 6500-7500), and product 2 (m/z 5000-6500).

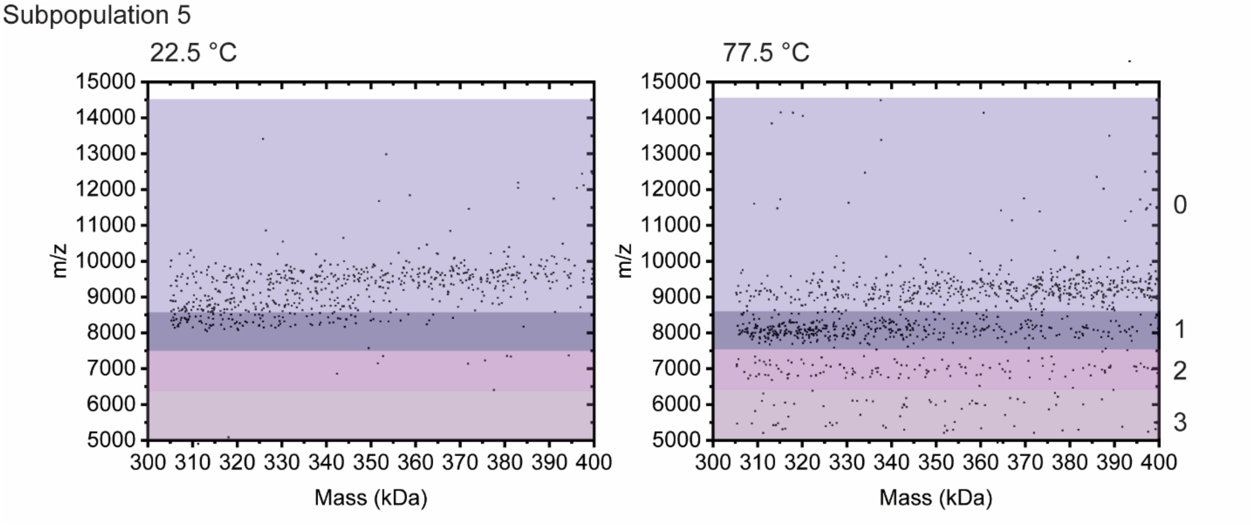

Figure S6. Scatter plot showing the mass-to-charge ratio (m/z) versus mass for every ion within subpopulation 5 at representative temperatures of (A) 22.5 °C and (B) 77.5 °C. Each point represents a single ion. Colored boxes represent m/z ranges for precursor (0, m/z 8500-14500), product 1 (m/z 7500-8500), product 2 (m/z 6500-7500), and product 3 (m/z 5000-6500).

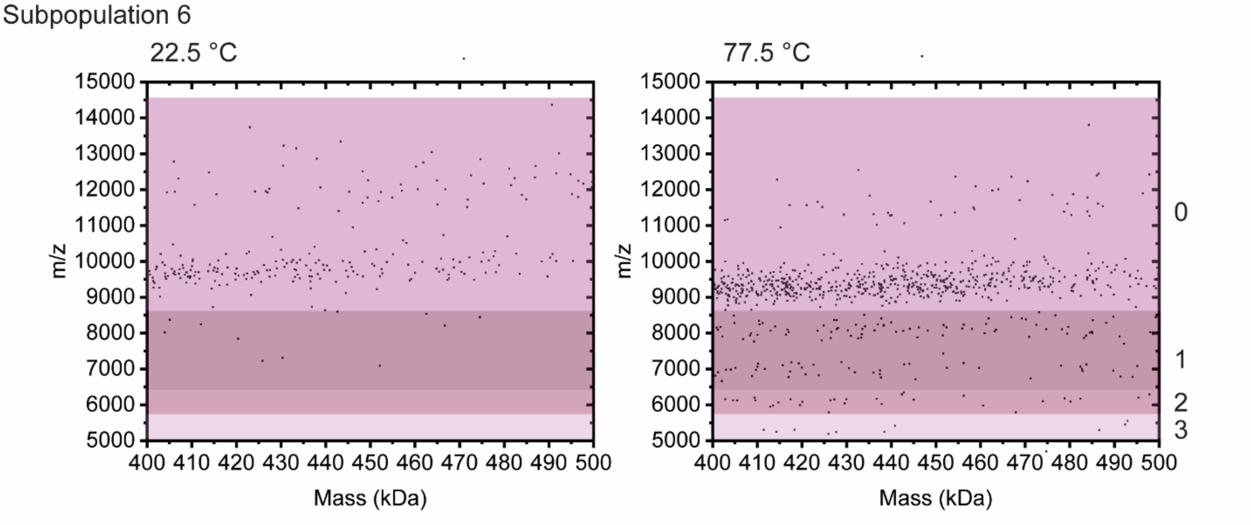

Figure S7. Scatter plot showing the mass-to-charge ratio (m/z) versus mass for every ion within subpopulation 6 at representative temperatures of (A) 22.5 °C and (B) 77.5 °C. Each point represents a single ion. Colored boxes represent m/z ranges for precursor (0, m/z 8500-14500), product 1 (m/z 6500-8500), product 2 (m/z 5700-6500), and product 3 (m/z 5000-5700).

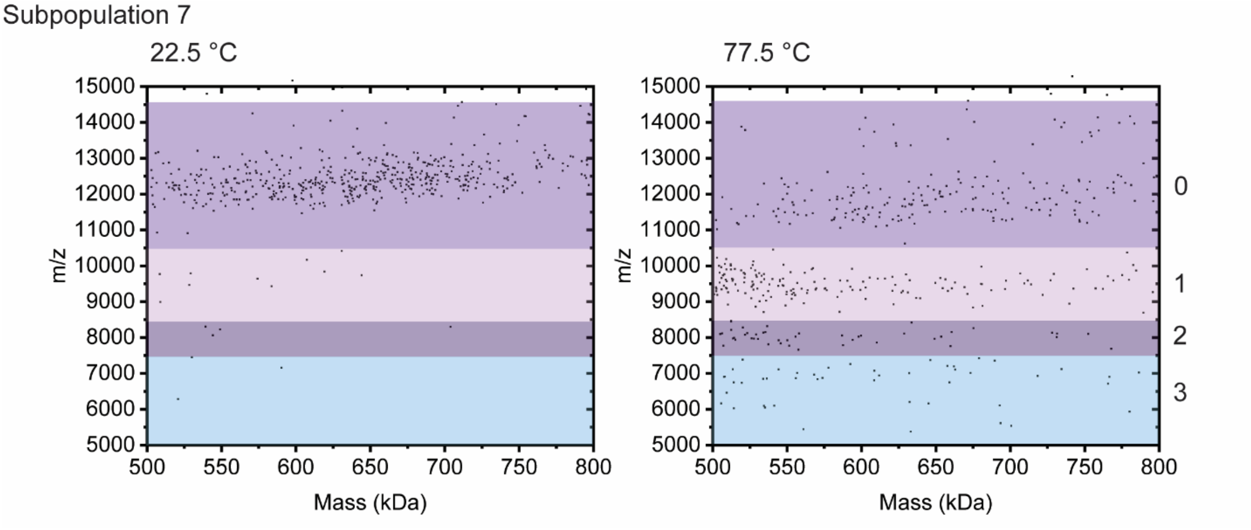

Figure S8. Scatter plot showing the mass-to-charge ratio (m/z) versus mass for every ion within subpopulation 7 at representative temperatures of (A) 22.5 °C and (B) 77.5 °C. Each point represents a single ion. Colored boxes represent m/z ranges for precursor (0, m/z 10500-14500), product 1 (m/z 8500-10500), product 2 (m/z 7500-8500), and product 3 (m/z 5000-7500).

Aggregation/Fusion

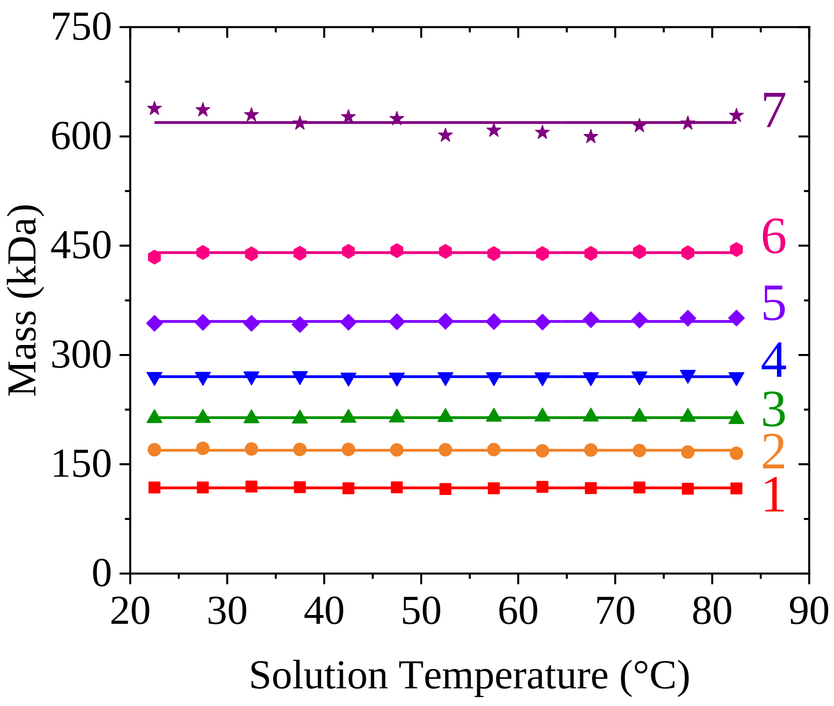

Figure S9. Plot of the average mass of subpopulations one through seven with respect to solution temperature. Each point represents the average mass of all ions within a mass window for each measured temperature. The solid lines represent the average mass for each subpopulation across all temperatures. Subpopulations one though seven have average masses of 117, 169, 214, 270, 346, 440, and 619 kDa, respectively.

References

[1] Jayaraman, S.; Gantz, D. L.; Gursky, O. Effects of Salt on the Thermal Stability of Human Plasma High-Density Lipoprotein. *Biochemistry*. **2006**, *45*, 4620-4628.

[2] Lu, M.; Gantz, D.L.; Herscovitz, H.; Gursky, O. Kinetic analysis of thermal stability of human low density lipoproteins: a model for LDL fusion in atherogenesis. *J Lipid Res*. **2012**, *53*, 2175–2185.

[3] El-Baba, T. J.; Clemmer, D. E. Solution thermochemistry of concanavalin A tetramer conformers measured by variable-temperature ESI-IMS-MS. Int. J. Mass Spectrom. 2019, 443, 93–100.

[4] Benesch, J. L. P.; Sobott, F.; Robinson, C. V. Thermal Dissociation of Multimeric Protein Complexes by Using Nanoelectrospray Mass Spectrometry. *Anal. Chem*. **2003**, *75*, 2208-2214.

[5] Wang, G.; Abzalimov, R. R.; Kaltashov, I. A. Direct Monitoring of Heat-Stressed Biopolymers with Temperature-Controlled Electrospray Ionization Mass Spectrometry. *Anal. Chem*. **2011**, *83*, 2870-2876.

[6] Cong, X.; Liu, Y.; Liu, W.; Liang, X.; Russell, D. H.; Laganowsky, A. Determining Membrane Protein-Lipid Binding Thermodynamics Using Native Mass Spectrometry. *J. Am. Chem. Soc*. **2016**, *138*, 4346-4349.
